# Focused Ultrasound in Neuroscience. State of the Art and Future Perspectives

**DOI:** 10.3390/brainsci11010084

**Published:** 2021-01-10

**Authors:** Giuseppe Roberto Giammalva, Cesare Gagliardo, Salvatore Marrone, Federica Paolini, Rosa Maria Gerardi, Giuseppe Emmanuele Umana, Kaan Yağmurlu, Bipin Chaurasia, Gianluca Scalia, Federico Midiri, Ludovico La Grutta, Luigi Basile, Carlo Gulì, Domenico Messina, Maria Angela Pino, Francesca Graziano, Silvana Tumbiolo, Domenico Gerardo Iacopino, Rosario Maugeri

**Affiliations:** 1 Neurosurgery Unit, Department of Biomedicine, Neurosciences & Advanced Diagnostics, School of Medicine, University of Palermo, 90127 Palermo, Italy; robertogiammalva@gmail.com (G.R.G.); salvo.mr89@gmail.com (S.M.); federicapaolini94@gmail.com (F.P.); rosamariagerardimd@gmail.com (R.M.G.); lbasile64@libero.it (L.B.); carlogul@yahoo.it (C.G.); messina.dtp1986@gmail.com (D.M.); mariangelapino@live.it (M.A.P.); gerardo.iacopino@gmail.com (D.G.I.); rosario.maugeri1977@gmail.com (R.M.); 2 Section of Radiological Sciences, Department of Biomedicine, Neurosciences & Advanced Diagnostics, School of Medicine, University of Palermo, 90127 Palermo, Italy; cesare.gagliardo@unipa.it (C.G.); federico.midiri@unipa.it (F.M.); 3 Department of Neurosurgery, Cannizzaro Hospital, 95126 Catania, Italy; 4 Departments of Neuroscience and Neurosurgery, University of Virginia Health System, Charlottesville, VA 22903, USA; ky7zb@virginia.edu; 5 Department of Neurosurgery, Neurosurgery Clinic, Birgunj 44300, Nepal; trozexa@gmail.com; 6 Department of Neurosurgery, Highly Specialized Hospital of National Importance “Garibaldi”, 95122 Catania, Italy; gianluca.scalia@outlook.it (G.S.); fragraziano9@gmail.com (F.G.); 7 Department of Health Promotion, Mother and Child Care, Internal Medicine and Medical Specialties-ProMISE, University of Palermo, 90127 Palermo, Italy; lagruttaludovico@gmail.com; 8 Division of Neurosurgery, Villa Sofia Hospital, 90146 Palermo, Italy; tumbiolosilvan@yahoo.it

**Keywords:** tcMRgFUS, HIFU, LIFU, focused ultrasound, neuro-oncology, neurodegenerative diseases, epilepsy, psychiatric disorders, blood-brain barrier

## Abstract

Transcranial MR-guided Focused ultrasound (tcMRgFUS) is a surgical procedure that adopts focused ultrasounds beam towards a specific therapeutic target through the intact skull. The convergence of focused ultrasound beams onto the target produces tissue effects through released energy. Regarding neurosurgical applications, tcMRgFUS has been successfully adopted as a non-invasive procedure for ablative purposes such as thalamotomy, pallidotomy, and subthalamotomy for movement disorders. Several studies confirmed the effectiveness of tcMRgFUS in the treatment of several neurological conditions, ranging from motor disorders to psychiatric disorders. Moreover, using low-frequencies tcMRgFUS systems temporarily disrupts the blood–brain barrier, making this procedure suitable in neuro-oncology and neurodegenerative disease for controlled drug delivery. Nowadays, tcMRgFUS represents one of the most promising and fascinating technologies in neuroscience. Since it is an emerging technology, tcMRgFUS is still the subject of countless disparate studies, even if its effectiveness has been already proven in many experimental and therapeutic fields. Therefore, although many studies have been carried out, many others are still needed to increase the degree of knowledge of the innumerable potentials of tcMRgFUS and thus expand the future fields of application of this technology.

## 1. Introduction

The employment of acoustic energy for diagnostic purposes represented 20th century medicine as the achievement of a great goal. The US allowed bridging the complicated gap between semiotics and pathology, defined by the need for more precise diagnostics tools. Sound waves are elastic mechanical waves that can only propagate in a “non-empty” space or a medium. The human ear has a limited ability to perceive sound waves between 20 and 20,000 Hz, and higher frequency waves are called ultrasounds. Ultrasounds are created and received by transducers; they are constituted of piezoelectric crystals that produce ultrasound beams when electrically charged. Some physical parameters are characteristic of a specific medium influence ultrasound delivery; these are propagation speed (which depends on the density and compressibility of the medium) and the acoustic impedance (which measures the forces that oppose the transmission of waves). These parameters vary from medium to medium and, therefore, from tissue to tissue. From the interaction between ultrasound beams and cells, four main physical phenomena occur reflection, refraction, diffusion, absorption. In the last few decades, many studies showed how ultrasounds could be used not only for clinical or intraoperative diagnostic purposes also for therapeutics [1].

Using ultrasounds for therapeutic purposes dates back to the early 1940s when Lynn and Putnam first performed targeted, well-demarcated and controlled ablations in cortical/subcortical areas of mammals [2]. In the 1950s, the Fry brothers used high-intensity ultrasounds to produce targeted necrotic lesions into brain basal ganglia of previously craniotomized animals without affecting the neighboring brain parenchyma [3]. At this former stage, a craniotomy was inevitable to convey focused ultrasound directly into brain parenchyma. With the development of devices consisting of multiple phased-array transducers, it was possible to deliver enough thermal energy in a small and sharp focus through the intact skull directly to deep brain structures to induce a controlled thermal ablation [4]. The introduction of MRI proton resonance frequency (PRF) shift thermometry, which is employed for monitoring the energy release in the targeted area and surrounding tissues, along with the possibility to precisely modulate the delivery of acoustic energy, made tcMRgFUS brain ablation a more accurate and safe technique, paving the way for the such called non-invasive functional neurosurgery [5].

During tcMRgFUS treatments for functional neurological disorders, the patient is awake and compliant in order to receive quick feedback on his health state and perform a neurological evaluation at the end of each sonication. Although tcMRgFUS procedures were initially conducted with three Tesla MRI scanners, today it has been demonstrated that they can be safely and effectively also performed with widely used 1.5 Tesla MRI scanners [4,6,7], which enables a high-resolution intraoperative imaging thanks to a dedicated MR coil still not available for 3T integrated tcMRgFUS systems [8,9].

The procedure is performed by applying a stereotactic frame to the patient’s head after a full scalp shaving. During tcMRgFUS ablative procedures, the patient lays inside the MRI scanner [10]. Pre-operative and intra-operative brain MRI allows for a precise calculation of stereotactic coordinates of the target and to evaluate the lesion appearance made by FUS. Initial low-energy sonications are used to assess the thermal map, confirming the absence of areas of unwanted overheating outside the target point, and to ensure the focusing of ultrasound beams into the target area [11,12]. Once the focusing is confirmed, the energy is gradually increased. During this stage, the patient is continuously monitored and neurologically evaluated after every sonication. Once the target is even clinically confirmed, with the best neurological improvement without adverse events, sonication parameters (temperature (C/F), frequency (Hz), application duration (sec), power (W), energy (J) are adjusted to reach a peak temperature above 52 °C, which makes a permanent necrotic lesion [12,13].

Unlike other ablative techniques such as stereotaxic radiosurgery, tcMRgFUS does not require radiations, thus avoiding the possible side effects such as brain edema, headache, aseptic meningitis, actinic keratosis, and alopecia. Moreover, it is performed over a short hospitalization, and it could even be repeated [14,15,16,17,18].

In recent years, tcMRgFUS has caused a great reverberation within the scientific community for its remarkable applications in treating movement disorders such as drug-resistant parkinsonian tremor and essential tremor, a psychiatric disorder such as major depressive disorder (MDD) and obsessive-compulsive disorder (OCD) through non-invasive ablative procedures. Moreover, recent interest has been inspired by the capability of focused ultrasounds to produce targeted transient alterations of the blood-brain barrier (BBB) and the subsequent possible employment in the treatment of oncological pathologies by improving the permeability of BBB to specific chemotherapy and by altering some of their biomolecular mechanisms [19,20,21].

The purpose of this study is to investigate potential future applications of tcMRgFUS and to enlighten the prospective field of employment and procedures in which tcMRgFUS has just been adopted.

## 2. Materials and Methods

An extensive search of English literature was performed on PubMed (https://pubmed.ncbi.nlm.nih.gov) using the following keywords and their combinations: FUS, Blood-Brain-Barrier, thalamotomy, essential tremor, Parkinsonisms, movement disorders, capsulotomy, psychiatric disorders, trigeminal neuralgia, Guillan-Barrè Syndrome (GBS), neuro-oncology. Preclinical and clinical studies of the last four years were carefully reviewed, focusing on new perspectives in focused ultrasounds. Publication time was restricted to the last four years to achieve a careful insight on future perspectives of tcMRgFUS. English publications with available full text were included along with their most meaningful references. Studies dealing with the comparison between tcMRgFUS and different treatments in the same pathology were also included.

Exclusion criteria were: DBS and gamma-knife radiosurgery procedures exclusively treated publication dated over ten years, unavailability of full text, non-English publications, publications in which previously mentioned queries were not related to tcMRgFUS, publications in which clinical conditions were not treated by tcMRgFUS, or publications in with previously mentioned clinical conditions without any comparison to tcMRgFUS. Studies focusing on essential tremor were excluded since the effectiveness of this treatment has been already confirmed and accepted. Inclusion and exclusion criteria are summarized in Table 1.

Then, the results of our queries were screened according to the PRISMA statement. Recent tgMRgFUS applications in neurosurgery were described from the included studies, and they were systematically organized and grouped in perspective.

## 3. Results

From the literature search performed on PubMed with the abovementioned queries, we identified 96 unique articles. These were later screened for relevance: 42 studies were excluded according to our exclusion criteria, and 54 were included according to our inclusion criteria. Full-text was available for all of 54 included studies, which were included in our qualitative analysis.

## 4. Discussion

TcMRgFUS is a novel technology that has been recently introduced in neurosurgery for the treatment of movement disorders. Beyond its proven effectiveness in functional neurosurgery, tcMRgFUS has recently shown several potential employment fields, and other prospective applications have just been being explored. For this review, recent and upcoming applications of tcMRgFUS have been summarized from the available literature and grouped for fields of applications.

### 4.1. Neuromodulation

Since 1962, it has been shown that mechanical stimulation of a peripheral axon can trigger action potentials, and it can interfere with the state of membrane-cell channels [22]. It has recently been demonstrated that ultrasonic stimulation onto the peripheral nerve can produce muscular contraction through action potentials’ modulation [23]. This phenomenon may be attributed to a transient deformation of the axon sheath by mechanical pressure of sound waves and the subsequent ionic stream through channels in Ranviers’ nodes. In fact, it has been demonstrated that focused ultrasound may produce transient focal alterations of brain circuits that are also noticeable through electroencephalography [24,25] or focal neural disconnection by the delivery of neurotoxin through permeabilized BBB [26]. In this way, low intensity focused ultrasounds (LIFU) (1–100 mW/cm2) may be used to excite or inhibit targeted clusters of neurons, to modulate nerve conduction, to modulate ionic stream, to promote neuroplasticity, to stimulate and further to non-invasively map the cerebral cortex, the deep brain nuclei and the white matter tracts [27,28,29,30,31,32,33,34,35,36,37].

### 4.2. Drug-Resistant Epilepsy

Recently tcMRgFUS has shown potential effectiveness in the treatment of drug-resistant epilepsy. About 20–40% of epileptic patients are affected by drug-resistant epilepsy, and they often undergo surgical treatment. Non-resective neuromodulatory treatments are performed by deep brain stimulation (DBS), vagus nerve stimulation (VNS), or responsive neurostimulation (RNS) [38].

Regarding the ablative surgery, it has been reported the first case of a female patient affected by mesial temporal lobe epilepsy undergone tcMRgFUS ablation. After the treatment, the patient was seizure-free and improved her quality of life at 1-year follow-up. Besides ablative applications, tcMRgFUS can modulate neurological functions by using low-frequency stimulation. Experiences are limited to preclinical studies; few clinical trials are still ongoing [39].

### 4.3. Drug-Resistant Trigeminal Neuralgia

Drug-resistant trigeminal neuralgia is a debilitating pain condition that often is treated by surgery. In such cases, recently, it has been shown that tcMRgFUS bilateral central-lateral thalamotomy effectively treats patients affected by drug-resistant trigeminal neuralgia, with 63% of good outcomes at three months, 88% at 1 year and 100% at last follow-up with no adverse events [40].

### 4.4. Chronic Neuropathic Pain

There is a lowering of the excitability threshold in chronic pain. Lower intensity stimulations suffice to activate spikes and transmit the painful signal along with dedicated neurological pathways.

It has been recently shown in a preclinical study that pulsed FUS on dorsal root ganglia may be an effective treatment of chronic neuropathic pain since it can determine a temporary reduction of mechanical allodynia secondary to an iatrogenic lesion of rat peroneal nerve [41]. The physiological mechanisms which could justify temporary analgesia are still unclear. However, it could be associated with the mechanical change of the ionic channels induced by acoustic waves and improved microcirculation and nerve conduction velocity. It has been found in a preclinical model of diabetic neuropathy [42].

Regarding clinical applications of focused ultrasound, it has been recently demonstrated that tcMRgFUS postero-central lateral thalamotomy is an effective treatment of drug-resistant chronic neuropathic pain and trigeminal neuralgia [40]. In contrast, local MRgFUS ablation effectively treats pain related to lumbar bone metastases [43,44].

### 4.5. Psychiatric Disorders

Patients affected by obsessive-compulsive disorder (OCD) and major depressive disorder (MDD) may be refractory to pharmacotherapy and cognitive-behavioral treatment. In case of refractory OCD, capsulotomy is the most effective procedure to control OCD symptoms. Capsulotomy can be performed by several techniques, such as radiofrequency, radiosurgery, and DBS. Among these, tcMRgFUS is an effective procedure aimed to disrupt hyperactivated circuits between the dorsal thalamus and prefrontal cortex. It is possible through precise targeting guided by MRI real-time monitoring, avoiding the exposure to ionizing radiation and radiation side effects [45,46,47,48].

In 2014, for the first time, four patients affected by medically refractory OCD were successfully treated by tcMRgFUS; no side effect was encountered after a six-month follow up [49]. As regards MDD, several brain structures may be identified as therapeutic targets. Drug refractory MDD is treated by anterior cingulotomy, subcaudate tractotomy, limbic leucotomy, and anterior capsulotomy [50]. It has recently been reported that tcMRgFUS bilateral anterior capsulotomy is an effective treatment of drug-resistant MDD in a 56-years-old patient, capable of alleviating symptoms and improving neuropsychological tests and quality of life [51].

### 4.6. Ischemic and Hemorrhagic Stroke 

Despite the incidence of ischemic and hemorrhagic stroke, only a fraction of patients benefit from medical (thrombolysis) and surgical treatment (clot evacuation/decompressive craniectomy) [52].

The implementation of ultrasounds in the treatment of ischemic stroke dates back to 2000, when transcranial doppler ultrasonography was used to monitor and improve the rate of medical thrombolysis [53]. It has been hypothesized that TcMrgFUS could further enhance the treatment of ischemic and hemorrhagic strokes through the targeted delivery of acoustic energy to the clot, thus performing the so-called “sonothrombolysis”. In 2013 and then in 2014, the effectiveness of tcMRgFUS sonotrhombolysis was demonstrated in the in-vivo porcine model and ex-vivo human model of intracranial hemorrhage. In these models, sonications were used to liquefy clot while MRI controlled treatment targeting and temperature monitoring. Clot liquefaction was achieved in both models between 2 and 24 h after clot formation [54,55]. Recently, promising results have been obtained by the adjunct of microbubbles to tMRgFUS sonotrombolysis in ischemic stroke, even the effectiveness needs to be confirmed by further in-vivo studies [56].

### 4.7. Modulation of Blood-Brain Barrier Permeability

In the 1970s, it has been described as the possibility of altering the BBB permeability by hypertonic solutions [57]. In recent years, it has been explored the potential to modulate BBB permeability by the use of pulsatile LIFU. In fact, while high-intensity ultrasound (1000 W/cm2) can induce coagulative necrotic lesions in targeted regions of the brain, pulsatile low-intensity ultrasound can interfere with BBB permeability without causing permanent lesion. Oscillations induced by pulsed LIFU result from the compression-release cycle and they would be able to alter the permeability of BBB tight junctions, transiently undocking tight bonds between endotheliocytes and then open that portion of the barrier (lasting about 4–6 h) [57]. One clinical study performed pulsated LIFU on 21 GBM patients who are under carboplatin treatment and concluded that pulsated LIFU might increase the drug therapies without inducing neurotoxicity [58]. In particular, BBB permeability would be mostly influenced by short pulse lengths and low pulse repetition frequencies of focused ultrasounds [59]. The evidence that LIFU can alter BBB permeability recently paved the way to new diagnostic and therapeutic possibilities. LIFU has been used for diagnostic purposes to obtain liquid biopsy in a mouse model of glioblastoma (GBM) [60,61]. In this case, the localized BBB opening induced by LIFU could increase vascular permeability and, therefore, the outflow of tumoral markers into the bloodstream and thus their detection [60,61]. Moreover, it has been demonstrated in a rat model of GBM that the adjunct of sonosensitizers such as 5-aminolevulinic acid could determine localized cytotoxic effects even in absence of thermal effects with the so called sonodynamic therapy [60,61].

Regarding the therapeutic application of LIFU, several other studies have been conducted in oncological and neurodegenerative diseases. In particular, it has been demonstrated that LIFU-induced BBB permeabilization could be enhanced by the administration of microbubbles [62], and this technique may be effective in improving chemotherapy delivery through BBB for the treatment of brain tumors [63,64,65]. The evidence of biochemical dyes extravasation confirmed the effectiveness and safety of microbubble administration in increasing BBB permeability during LIFU treatment from one to 96 h after treatment in the absence of lesions. This evidence paves the way to obtaining a microbubble-mediate chemical treatment by inserting drug moles inside nanoparticles during LIFU treatment to obtain the localized emission of the compound to the targeted tissue, thus maximizing the local concentration and reducing systemic toxicity. This technique has been named “sonopermeation” [62,66]. The combination of LIFU and high intensity focused ultrasounds (HIFU) recently implemented microbubble BBB permeabilization to alter BBB’s permeability and subsequently convey poly (2-ethyl-butyl cyanoacrylate) nanoparticle-stabilized microbubbles through the extracellular matrix of a rat model of melanoma brain metastasis. Further microscopic studies underlined in some animals a directly proportional correlation between the rate of BBB permeabilization and the distribution of nanoparticles [67]. This technique presents several limitations. One of them is related to the range of effective sonication parameters used to obtain a therapeutic effect and acceptable impact on tissue health, avoiding complications such as hemorrhage, edema, extensive inflammation, ischemia, apoptosis, prolonged BBB permeabilization. Another important limitation is related to the correct dose of microbubble to infuse. Several factors that can influence how microbubbles respond to ultrasounds, and there are no standard protocols in these procedures. Moreover, inhomogeneities in BBB can lead to different effects related to a different location. In particular, transient enhancing in BBB permeability can lead to increased complication rate [62,66].

While clinical applications of tcMRgFUS for localized chemotherapy delivery through BBB sonopermeation is still controversial [68], several studies have already enlightened a localized pro-inflammatory activity of tcMRgFUS due to the stimulation of innate and acquired immune responses through local immunomodulation and gene-regulation [69,70]. This paves the way to novel frontiers to treat brain tumors and the treatment of neurodegenerative diseases through gene-delivery therapy. Sonoselective gene-delivery has recently been possible through endothelial-sonoselective transfection of the cerebral blood vessels without opening BBB without inducing sterile inflammation. This novel technique could promote angiogenesis, releasing nerve growth factors, and promoting neurogenesis through the recruitment of stem cells [71].

Regarding new perspectives in the treatment of neurodegenerative diseases, the delivery of a gene-liposome system containing glial cell line-derived neurotrophic factor has been recently possible by applying pulsed FUS with microbubbles in a transgenic mouse model of Huntington disease. This technique has been possible to obtain an effective gene therapy with reduced polyglutamine-expanded aggregates and improved motor function [72].

Based on the evidence of BBB dysfunction since the early stage of neurodegenerative diseases [73,74], it has been recently possible to demonstrate that a targeted gene and protein therapy in combination with FUS sonopermeation and microbubbles is capable of attenuating the nigrostriatal pathway degeneration since the early stage in a mouse model of Parkinson Disease (PD) [75].

As regards current clinical scenarios, tcMRgFUS sonopermeation has recently been used to induce a transitory BBB permeation localized at the primary motor cortex of some ALS volunteers, without clinically and electrophysiologically noticeable complications, opening up the possibility of targeted therapy such as localized delivery of superoxide dismutase (SOD) inhibitors [76]. Previously, the possibility of reducing up to 20% of beta-amyloid plaques has been already described in a rat model of Alzheimer Disease (AD) undergone tcMRgSUS sonopermeation for targeted delivery of exogenous antibodies [77,78]. The local effect of sonopermeation on AD beta-amyloid plaques would be further increased by the BBB crossing of endogenous antibodies from the systemic bloodstream and the local activation of microglia [79]. Since microglial activation, the more significant beta-amyloid could be internalized in lysosomes, leading to improved memory functions in mouse models of AD [80]. This virtuous circle could be probably sustained also by enhanced kinase inhibitors (i.e., GSK-3 inhibitor) delivery through a permeabilized BBB [81].

In conclusion, the whole backdrop of FUS application in BBB modulation and neurodegenerative disease treatment is a very prospective study field. Nowadays, several other applications are still to be tested.

### 4.8. Progression in Neuro-Oncology

The neuro-oncological field experiences are limited to small studies in patients with no surgical treatment possibility [82]. The thermal effect of HIFU, according to temperature, can achieve both ablative effect (55–60 °C or 240 CEM 43°, cumulative equivalent minutes at 43 °C) and radiosensitization induced by mild hyperthermia [83].

Moreover, the release of tumor antigens induced by necrosis can enhance antitumor immunity. Heat generation arises from sonication and cavitation. Cavitation also has a mechanical effect, essential for BBB disruption [84].

In this regard, the first study was a Phase I/II study with untreatable patients affected by recurrent malignant gliomas. They underwent a craniectomy and a biopsy of the tumor; 7–10 days later, a tcMrgFUS treatment was performed. The first patient had a tumor volume of 9 cm^3^; sonications did not achieve ablation temperature because of attenuation by synthetic dural substitute implanted during previous operations. The second patient had a successful ablation of 50% of tumor volume; after the procedure, no radiological or neurological adverse events were noticed, apart from a transient worsening of her pre-existing dysphasia that improved with steroid therapy. She has undergone surgery to complete removal, and histological analysis showed necrosis induced by FUS. The third patient had a complete ablation of all 5cm^3^ tumor volume; a 24 h MRI showed a new lesion in the right cerebral peduncle, with no clinical findings, resolving spontaneously. He had a recurrence after four months [85]. The first successful tcMRgFUS ablation of brain tumor was reported in a 17-year-old female affected by non-surgical anaplastic astrocytoma, whose volume decreased six months after tcMRgFUS treatment [86].

First sonic ablations without previous craniectomy were attempted in 2010. Treatments were performed in three patients with glioblastoma, not eligible for the surgical procedure, who previously underwent chemotherapy and radiotherapy. Unfortunately, they did not reach sufficient US power to achieve ablation due to skull characteristics [87].

The first successful non-invasive thermal ablation using tcMrgFUS was performed in 2014. The procedure allowed physicians to ablate 10% of tumor volume; however, time consumption and overheating of the skull represented two of the most critical limits of the procedure [88].

Based on these studies, tcMRgFUS seems to be a feasible treatment option for brain tumor ablation, but other studies and clinical experiences are still needed. In the attempt to achieve a total ablation of lesions, three Phase I trials are still ongoing [89,90,91].

## 5. Conclusions

MRgFUS is a versatile and promising technique whit several and still undiscovered applications in diagnostic and therapeutic fields. However, since its several areas of applications, more preclinical and clinical studies are still needed. To sum up, tcMRgFUS shows several ambitious applications and new potential capabilities that deserve continuous meticulous investigations. On the other hand, nowadays, tcMRgFUS procedures for functional neurological disorders affect the field as a valid non-invasive, safe, and effective therapeutic option.

## Figures and Tables

**Table 1:** Inclusion and exclusion criteria.

Inclusion Criteria	Exclusion Criteria
Publications of the last four years and their most meaningful references	Publications dated over four years
New perspectives in pathology treatment	Studies focusing on Essential Tremor
Availability of full text	Unavailability of full text
English publications	Non-English publications
Comparison between tcMRgFUS and different treatments in the same pathology	Studies without any comparison to tcMRgFUS
